# The Δ4-desaturation pathway for DHA biosynthesis is operative in the human species: Differences between normal controls and children with the Zellweger syndrome

**DOI:** 10.1186/1476-511X-9-98

**Published:** 2010-09-09

**Authors:** Manuela Martinez, Natalia Ichaso, Fernando Setien, Nuria Durany, Xiao Qiu, William Roesler

**Affiliations:** 1 Manuela Martinez Foundation for Children with Metabolic Diseases. Research Laboratory. Plaza Karl Marx 1, Barcelona 08042, Spain; 2 Cancer Epigenetics and Biology Program (PEBC), Av. Gran Vía s/n, km. 2.7 L'Hospitalet de Llobregat, Barcelona 08907, Spain; 3 International University of Catalunya, Department of Molecular and Cell Biology, Hospital General de Catalunya, Josep Trueta, s/n, Sant Cugat del Vallès 08195, Spain; 4 Department of Food & Bioproduct Science, University of Saskatchewan, 51 Campus Drive, Saskatoon, Saskatchewan S7N 5A8, Canada; 5 Department of Biochemistry, University of Saskatchewan, Saskatoon, Saskatchewan S7N 5E5, Canada

## Abstract

**Background:**

Docosahexaenoic acid (DHA, 22:6ω3) is a fundamental component of cell membranes, especially in the brain and retina. In the experimental animal, DHA deficiency leads to suboptimal neurological performance and visual deficiencies. Children with the Zellweger syndrome (ZS) have a profound DHA deficiency and symptoms that can be attributed to their extremely low DHA levels. These children seem to have a metabolic defect in DHA biosynthesis, which has never been totally elucidated. Treatment with DHA ethyl ester greatly improves these patients, but if we could normalize their endogenous DHA production we could get additional benefits. We examined whether DHA biosynthesis by Δ4-desaturation could be enhanced in the human species by transfecting the enzyme, and if this could normalize the DHA levels in cells from ZS patients.

**Results:**

We showed that the Δ4-desaturase gene (*Fad4*) from *Thraustochytrium sp*, which can be expressed by heterologous transfection in other plant and yeast cells, can also be transfected into human lymphocytes, and that it expresses the enzyme (FAD4, Δ4-desaturase) by producing DHA from direct Δ4-desaturation of 22:5ω3. We also found that the other substrate for Δ4-desaturase, 22:4ω6, was parallely desaturated to 22:5ω6.

**Conclusions:**

The present "in vitro" study demonstrates that Δ4-desaturase can be transfected into human cells and synthesize DHA (as well as 22:5ω6, DPA) from 22:5ω3 and 22:4ω6, respectively, by putative Δ4-desaturation. Even if this pathway may not be the physiological route for DHA biosynthesis "in vivo", the present study opens new perspectives for the treatment of patients within the ZS spectrum.

## Background

Docosahexaenoic acid (DHA, 22:6ω3) is a polyunsaturated fatty acid (PUFA) of fundamental importance in cell membranes, especially in nerve endings and the photoreceptor cells in the retina [1,2]. DHA is considered essential during brain development, especially after 31 weeks of gestation, when its accretion is maximal [3]. In the clinical setting, DHA deficiency has been related to several human diseases [4].

In the human species, it is generally agreed that synthesis of DHA from its essential precursor 18:3ω3 (α-linolenic acid) is inadequate, especially in the premature infant, where it does not cover the daily needs of this important PUFA. Because of that, DHA is being added to many infant formulas during the last years. Even in the adult, DHA is being increasingly recommended to improve several conditions, with more or less convincing basis and results. Thus, based on its marginally decreased levels, DHA is currently being recommended as a supplement for such varied diseases as attention deficit hyperactivity disorder [5], fetal alcohol syndrome [6], phenylketonuria [7], schizophrenia [8], unipolar depression [9], aggressive behavior [10], Alzheimer's disease [11] and diabetes [12]. However, it is only in one group of diseases -the Zellweger syndrome and its related phenotypes- where the DHA levels are dramatically diminished in all tissues, including the brain and retina [13,14].

Generalized peroxisomal disorders within the Zellweger syndrome spectrum, usually called *peroxisomal biogenesis disorders *(PBD), are lethal congenital diseases, characterized by the lack of functional peroxisomes in the cells of the body [15]. As a consequence, several biochemical reactions related to lipid metabolism, which normally occur in the peroxisome, are affected. Among them, β-oxidation of pristanic and very long chain fatty acids (VLCFA) [16], as well as α-oxidation of phytanic acid [17], are defective. Biosynthesis of plasmalogens [18] and biliary acids is also defective [19]. In addition, we found that these children show a profound DHA deficiency [13,14]. In classic Zellweger's syndrome (ZS), the prototype of these disorders, brain DHA levels may be so low as to only account for 20-25% of the normal values [3,13], and in the Zellweger retina, DHA is virtually absent [14].

Clinically, these patients become blind, deaf, and mentally retarded very early in life. Myelin is always defective in classic Zellweger's syndrome (ZS), in both amount and quality (dysmyelination), and liver function is consistently impaired. Renal cysts are commonly added, which gave the name of cerebro-hepato-renal syndrome to the first ZS patients described [20,21] The life span of these patients is very short, especially in classic ZS, where survival rarely surpasses the first year of age. There are milder Zellweger variants, known as neonatal adrenoleukodystrophy (NALD) and infantile Refsum's disease (IRD). These relatively milder phenotypes, however, present most of the symptoms of classic ZS, including profound mental retardation with myelination delay, followed later by demyelination, visual and hearing sensorineural defects and liver involvement. There is no means to differentiate the various ZS phenotypes, when based on genotype or biochemical abnormalities alone. They are mainly clinical entities, with extremely variable organ involvement and prognosis. Without a treatment and special care, however, all these patients live very short lives, rarely reaching older childhood. In spite of that, correcting their DHA deficiency produces substantial benefits in their myelination [22], liver function [23] and vision [24], thus prolonging survival and quality of life markedly. We believe that DHA deficiency plays a crucial role in this group of congenital disorders, as most of the patients' symptoms are consistent with what is found in DHA-deficient animals [25] and humans [26]. ZS patients - even more than normal children - are probably incapable of producing all the DHA required by the fast development of their brain and retina.

There is some controversy about the mechanism of DHA biosynthesis in mammals. Classically, it was accepted that DHA is formed by front-end, putative Δ4-desaturation of its immediate precursor 22:5ω3, by introducing a double bond in position Δ4 (counting from the carboxyl end, IUPAC nomenclature). In parallel, this enzyme would also convert 22:4ω6 to 22:5ω6 (ω6 docosapentaeoic acid, DPA). In lower eukaryotes, like the *Thraustochytrium sp*, the existence of a Δ4-desaturase has been demonstrated. When expressed in yeast (*Saccharomyces cerevisiae*) and plants *(Brassica juncea)*, this enzyme Δ4-desaturates 22:5ω3 to 22:6ω3 [27]. This results in the controlled production of docosahexaenoic acid, an interesting method for the nutraceutical industry, given the decreasing supply of DHA from natural, marine products.

In mammals, this pathway has practically been abandoned and an alternate route involving the peroxisome has been generally accepted [28]. This route would imply the microsomal elongation of 22:5ω3 to 24:5ω3, followed by a second Δ6-desaturation step to 24:6ω3, which would finally be β-oxidated in the peroxisome to produce DHA. Again, the same route would work for the ω6 family, finally yielding 22:5ω6 from 24:5ω6. Whether or not humans have an intrinsic Δ4-desaturase remains unknown. Nevertheless, the present paper shows for the first time that the Δ4-desaturation pathway is functional in the human species when cells are transfected with the enzyme. We show that when the gene that codes for this enzyme (*Fad4*) is transfected into human lymphocytes immortalized with the Epstein-Barr virus (EBV), putative Δ4-desaturation of both substrates, 22:5ω3 and 22:4ω6, is produced, yielding DHA and DPA, respectively, as their products.

## Results

### Transfection of the FlagΔ4 desaturase gene

**Figure 1:**
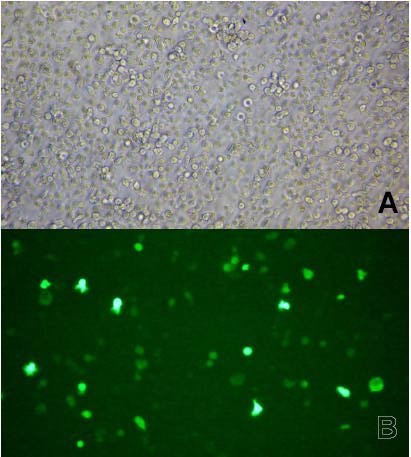
**Microscopic images of human immortalized lymphocytes transfected with the Δ4-desaturase vector**. Human B lymphoblastoid cells from one of the control cases, transiently transfected with the pIRES2EGFP-FlagΔ4 vector on day 3 after electroporation. Upper (A) and lower (B) panels show the cells under bright field and fluorescence microscopy, respectively.

As an example, Fig. 1 shows one of the control EBV immortalized lymphocyte lines, transiently transfected with the plasmid carrying the Δ4-desaturase gene (pIRES2EGFP-FlagΔ4 construct) on day 3 after electroporation. Upper (A) and lower (B) panels show the cells under bright field and fluorescence microscopy, respectively. By comparing both images and by direct counting of the cells in the various cases studied, we could estimate that the transfection efficiency was quite low, widely varying between 5 and 25%.

### Lymphocyte fatty acid composition

It must be pointed out that the fatty acid patterns are deeply altered when lymphocytes are immortalized with the EBV. Table 1 shows the main differences. Among PUFA, it can be seen that the proportion of arachidonic acid (AA, 20:4ω6) is much lower in EBV immortalized lymphocytes (as is the 20:4ω6/20:3ω6 ratio) than in the normal, circulating cells, whereas that of ω3 fatty acids is higher. This can be due to the multiple manipulations suffered by these cells and/or to the fetal calf serum used in the medium. In our experience, something similar occurs in human cultured skin fibroblasts, where the ω6/ω3 proportion is lower and the ratio 22:6ω3/22:5ω3 is very close to one. This is not what happens in fresh human cells, where DHA clearly predominates. As cultured cells, bovine serum is known to have lower ω6/ω3 and 22:6ω3/22:5ω3 ratios than human plasma and this can influence the composition of human cells nourished with it. However, the advantages of obtaining great amounts of immortalized lymphocytes by far outweigh this drawback. Besides, a high proportion of 22:5ω3 is an advantage when we want to know its possible conversion to DHA. Other marked differences, although probably of less biological importance, are the much higher percentage of monounsaturated fatty acids in EBV immortalized lymphocytes than in fresh cells. Omega-9, but also ω7 fatty acids, are many times higher in EBV immortalized lymphocytes than in fresh cells, something that we have also found in cultured skin fibroblasts (data not shown).

**Table 1:** Comparison between the main fatty acid composition of freshly obtained human lymphocytes and lymphoblastoid cell lines transfected with the empty vector.

	Fresh	EBV immortalized
14:0	0.49	2.06
16:0	19.59	21.06
16:1ω9	0.28	2.21
16:1ω7	0.28	1.27
18:0	19.72	15.66
18:1ω9	15.30	19.29
18:1ω7	1.68	7.29
18:2ω6	7.49	2.75
18:3ω6	0.03	0.09
18:3ω3	0.09	0.06
20:0	1.00	0.25
20:1ω9	0.64	1.45
20:3ω9	0.10	0.74
20:3ω6	1.65	3.21
20:4ω6	19.79	7.06
20:5ω3	0.31	0.58
22:4ω6	2.13	1.23
22:5ω6	0.37	0.31
22:5ω3	1.34	4.20
22:6ω3	2.01	4.46
ω6/ω3	7.72	1.41
22:6ω3/22:5ω3	1.50	1.06
22:5ω6/22:4ω6	0.17	0.26
20:4ω6/20:3ω6	11.99	2.20
22:4ω6/20:4ω6	0.11	0.17

This table compares the main fatty acid composition (mol%) of freshly processed human lymphocytes with that of lymphoblastoid cell lines immortalized with the Epstein-Barr virus and transfected with the empty plasmid pIRES2EGFP (mean). Note the very different arachidonic acid content of fresh and treated cells. In contrast, the ω3 PUFAs are higher and the 22:6ω3/22:5ω3 ratio is comparatively lower in the processed cells. Also, note the higher values of ω9 and ω7 fatty acids in the EBV-treated cells.

**Figure 2:**
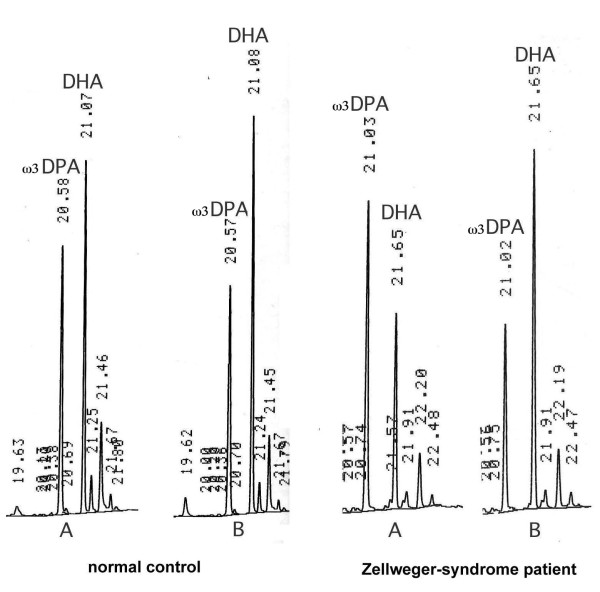
**Gas-chromatographic differences of the main omega-3 PUFAs between the normal and the Zellweger-syndrome child**. Omega-3 VLPUFA peaks directly scanned from the gas chromatograms in one control and one ZS patient. It can be seen that, before adding the Δ4-desaturase construct (A), DHA is higher than ω3DPA (22:5ω3) in the normal child, while it is lower than ω3DPA in the patient with Zellweger's syndrome (ratio 22:6ω3/22:5ω3 < 1.0) When adding the enzyme (B), this ratio increases clearly, especially in the ZS patient.

Fig. 2 visually shows the effects of transfecting the enzyme in a normal child and a patient with Zellweger's syndrome. Even by just looking at the gas chromatograms, it can be seen that Δ4-desaturation of the precursor 22:5ω3 (ω3DPA) to 22:6ω3 (DHA) always occurred in the gene-transfected cells (B) when compared to those transfected with the empty vector (A). However, this Δ4-desaturation effect was clearly greater in the Zellweger-syndrome patients than in normal children. Without adding the enzyme, the 22:6ω3/22:5ω3 ratio was usually greater than 1.0 in the normal controls, whereas in ZS patients, there was an inversion of the normal situation, DHA being lower than ω3DPA. After transfecting the Δ4-desaturation gene, however, all cells showed a 22:6ω3/22:5ω3 ratio clearly higher than 1.0. It must be emphasized that the changes observed were due to the transfected enzyme, not to any nutritional differences, the only DHA and ω3DPA added to the medium being those contained in the fetal calf serum, which was constant in all cases.

Table 2 shows the complete list of lymphocyte fatty acids in control and PBD patient cells, without the Δ4-desaturase gene. Table 3 shows the same two groups when the Δ4-desaturase gene was transfected. The statistical significance of the different fatty acids is shown. Only the mono and poly-unsaturated fatty acids change significantly between the two groups. As expected, the ratio of 22:6ω3 to 22:5ω3 was lower in the ZS patient group, at a very high level of statistical significance (p value lower than 0.0001). A similar change in the 22:5ω6/22:4ω6 ratio was observed, which was very significantly lower in the ZS patients than in controls.

**Table 2:** Fatty acid composition of lymphoblastoid cell lines transfected with the empty vector, in healthy controls and in patients with the Zellweger syndrome.

	Controls		Zellweger syndrome		
			
Fatty acid	Mean ± SEM	N	Mean ± SEM	N	Statistics
12:0	0.13 ± 0.011	16	0.27 ± 0.049	14	n.s.
14:0	2.06 ± 0.122	16	1.87 ± 0.101	14	n.s.
16:0	21.06 ± 0.343	16	21.58 ± 0.231	14	n.s.
16:1ω9	2.21 ± 0.100	16	2.03 ± 0.070	14	n.s.
16:1ω7	1.27 ± 0.082	16	1.13 ± 0.058	14	n.s.
18:0	15.66 ± 0.220	16	16.91 ± 0.315	14	P < 0.005
18:1ω9	19.29 ± 0.482	16	17.01 ± 0.761	14	P < 0.05
18:1ω7	7.29 ± 0.190	16	6.27 ± 0.275	14	P < 0.01
18:2ω6	2.75 ± 0.113	16	2.42 ± 0.076	14	P < 0.05
18:3ω6	0.09 ± 0.006	16	0.05 ± 0.015	14	n.s.
18:3ω3	0.06 ± 0.008	16	0.03 ± 0.009	14	n.s.
20:0	0.25 ± 0.010	16	0.27 ± 0.015	14	n.s.
20:1ω6	1.45 ± 0.071	16	1.66 ± 0.182	14	n.s.
20:2ω6	0.36 ± 0.017	16	0.42 ± 0.029	14	n.s.
20:3ω9	0.74 ± 0.048	16	0.65 ± 0.035	14	n.s.
20:3ω6	3.21 ± 0.108	16	2.64 ± 0.142	14	P < 0.005
20:4ω6	7.06 ± 0.328	16	8.36 ± 0.696	14	n.s.
20:5ω3	0.58 ± 0.042	16	0.49 ± 0.052	14	n.s.
22:0	0.32 ± 0.018	16	0.32 ± 0.022	14	n.s.
22:1ω9	0.25 ± 0.023	16	0.39 ± 0.028	14	P < 0.001
22:4ω6	1.23 ± 0.083	16	2.15 ± 0.168	14	P < 0.0001
22:5ω6	0.31 ± 0.018	16	0.25 ± 0.021	14	P < 0.05
22:5ω3	4.2 ± 0.218	16	4.88 ± 0.233	14	P < 0.005
22:6ω3	4.46 ± 0.173	16	3.91 ± 0.085	14	P < 0.01
24:0	0.41 ± 0.017	16	0.48 ± 0.021	14	P < 0.005
24:1ω9	0.94 ± 0.073	16	0.86 ± 0.078	14	P < 0.005
26:0	0.05 ± 0.009	16	0.14 ± 0.015	14	P < 0.0001
26:1ω9	0.15 ± 0.020	16	0.38 ± 0.044	14	P < 0.0001
24:4ω6	0.02 ± 0.007	16	0.03 ± 0.022	13	n.s.
24:5ω6	0.02 ± 0.007	16	0.14 ± 0.038	13	P < 0.005
24:5ω3	0.02 ± 0.008	16	0.06 ± 0.023	13	n.s.
24:6ω3	0.08 ± 0.014	16	0.15 ± 0.043	13	n.s.
22:6ω3/22:5ω3	1.07 ± 0.020	16	0.83 ± 0.046	14	P < 0.0001
22:5ω6/22:4ω6	0.28 ± 0.018	16	0.12 ± 0.011	14	P < 0.0001

This table is a list of the mean values ± SEM of total fatty acids (mol%) and their main ratios in human lymphocytes transfected with the empty plasmid (pIRES2EGFP), in normal controls as well as in Zellweger-syndrome patients. P values are only shown for statistically significant changes.

**Table 3:** Fatty acid composition of lymphoblastoid cell lines transfected with the Δ4-desaturase vector, in healthy controls and in patients with the Zellweger syndrome.

	Controls		Zellweger syndrome		
			
Fatty acid	Mean ± SEM	N	Mean ± SEM	N	Statistics
12:0	0.12 ± 0.021	16	0.24 ± 0.057	14	n.s.
14:0	1.75 ± 0.124	16	1.80 ± 0.103	14	n.s.
16:0	21.49 ± 0.239	16	21.58 ± 0.396	14	n.s.
16:1ω9	2.13 ± 0.096	16	1.99 ± 0.066	14	n.s.
16:1ω7	1.12 ± 0.077	16	1.12 ± 0.066	14	n.s.
18:0	15.60 ± 0.202	16	16.83 ± 0.326	14	P < 0.005
18:1ω9	18.45 ± 0.439	16	17.25 ± 0.756	14	n.s.
18:1ω7	7.09 ± 0.136	16	6.34 ± 0.265	14	n.s.
18:2ω6	2.73 ± 0.122	16	2.43 ± 0.085	14	n.s.
18:3ω6	0.11 ± 0.020	16	0.05 ± 0.015	14	n.s.
18:3ω3	0.06 ± 0.012	16	0.04 ± 0.009	14	n.s.
20:0	0.31 ± 0.050	16	0.27 ± 0.016	14	n.s.
20:1ω9	1.37 ± 0.071	16	1.69 ± 0.179	14	n.s.
20:2ω6	0.37 ± 0.019	16	0.39 ± 0.017	14	n.s.
20:3ω9	0.70 ± 0.065	16	0.64 ± 0.050	14	n.s.
20:3ω6	3.39 ± 0.083	16	2.68 ± 0.145	14	P = 0.0001
20:4ω6	7.69 ± 0.306	16	8.34 ± 0.681	14	n.s.
20:5ω3	0.56 ± 0.049	16	0.55 ± 0.073	14	n.s.
22:0	0.32 ± 0.023	16	0.30 ± 0.022	14	n.s.
22:1ω9	0.25 ± 0.017	16	0.39 ± 0.021	14	P < 0.0001
22:4ω6	1.19 ± 0.078	16	1.57 ± 0.139	14	P < 0.05
22:5ω6	0.56 ± 0.031	16	0.75 ± .053	14	P < 0.005
22:5ω3	3.58 ± 0.202	16	3.21 ± 0.157	14	n.s.
22:6ω3	5.56 ± 0.149	16	5.63 ± 0.159	14	n.s.
24:0	0.37 ± 0.011	16	0.47 ± 0.053	14	n.s.
24:1ω9	0.79 ± 0.072	16	0.86 ± 0.057	14	n.s.
26:0	0.06 ± 0.018	16	0.16 ± 0.029	14	P < 0.01
26:1ω9	0.11 ± 0.012	16	0.33 ± 0.027	14	P < 0.0001
24:4ω6	0.02 ± 0.008	16	0.01 ± 0.007	13	n.s.
24:5ω6	0.02 ± 0.011	16	0.11 ± 0.019	13	P = 0,0002
24:5ω3	0.03 ± 0.007	16	0.01 ± 0.005	13	P < 0.05
24:6ω3	0.07 ± 0.015	16	0.12 ± 0.010	13	P < 0.05
22:6ω3/22:5ω3	1.62 ± 0.096	16	1.82 ± 0.129	14	n.s.
22:5ω6 22:4ω6	0.52 ± 0.048	16	0.53 ± 0.064	14	n.s.

This table lists the mean values ± SEM of total fatty acids (mol%) and their main ratios in immortalized lymphocytes transfected with the Δ4-desturase plasmid (pIRES2EGFP-FlagΔ4), in normal controls as compared with Zellweger-syndrome patients. As in table 2, p values are only shown for statistically significant changes.

**Figure 3:**
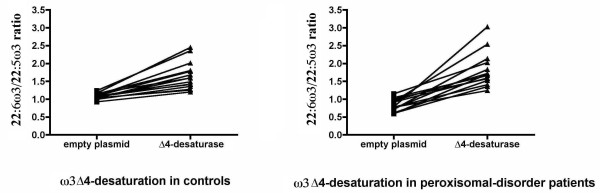
**Omega-3 individual effects of transfection with Δ4-desaturase on controls and children with the Zellweger syndrome**. This figure shows the significant increases of the 22:6ω3/22:5ω3 ratio in all the children transfected with the Δ4-desaturase plasmid. As can be seen, the ratio increases clearly in all the cases. However, the slope is higher in the ZS patients, whose ratio is lower from the start and surpasses the normal in some patients. The greater variability in the ZS patients is due to their clinical and biological heterogeneity. Yet, the statistical significance of paired t-tests is extremely high in all cases (p < 0.0001)

**Figure 4:**
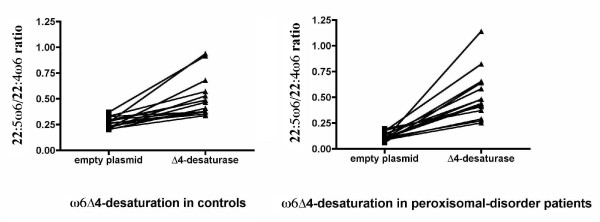
**Omega-6 individual effects of transfection with Δ4-desaturase on controls and children with the Zellweger syndrome**. This figure shows the significant increases in the 22:5ω6/22:4ω6 ratio in all the children transfected with the Δ4-desaturase vector. As can be seen, the ratio increases clearly in all the cases (p value lower than 0.0001 by paired t-tests). Again, the slope is higher in the ZS patients, who start with lower ratios than the controls. Here too, the ZS patients show a greater variability in the ratio, although the statistical significance remains extremely high.

Fig. 3 and 4 show these ratios individually. It can be seen that both ratios increased when the Δ4-desaturase gene was epitopically expressed. Despite the low transfection efficiency, there was no case without a response of both ω3 and ω6 PUFA precursors. Again, the ZS patients showed a greater effect of the enzyme. This effect was variable due to the wide phenotype heterogeneity, as well as the variations inherent to the methods used. Nevertheless, the enzyme action was evident even more clearly in the patients than in controls, with all the changes showing an extremely high statistical significance (p < 0.0001) by paired t-tests. In most cases, the 22:5ω6/22:4ω6 ratio increased even more than the 22:6ω3/22:5ω3 ratio, although its variability was higher. This supports the hypothesis that Δ4-desaturase is a common enzyme in this pathway, by introducing a double bond in 22:5ω3, as well as in 22:4ω6.

**Figure 5:**
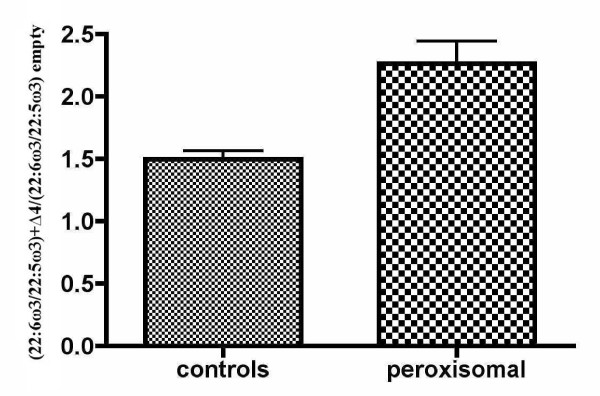
**Omega-3 effects of transfection with Δ4-desaturase on grouped controls and patients with the Zellweger syndrome**. The number of times that the 22:6ω3/22:5ω3 ratio increased when the Δ4-desaturase construct was transfected, in normal children and in ZS patients, is displayed here. For that, a new index was obtained by dividing the 22:6ω3/22:5ω3 ratio with the added Δ4-desaturase by the same ratio obtained when transfecting the empty vector [(22:6ω3/22:5ω3)+Δ4/(22:6ω3/22:5ω3) empty]. Bars are mean values ± SEM. Despite the effect of grouping patients and controls by unpaired t-tests, the statistical significance was still extremely high (p = 0.0002).

**Figure 6:**
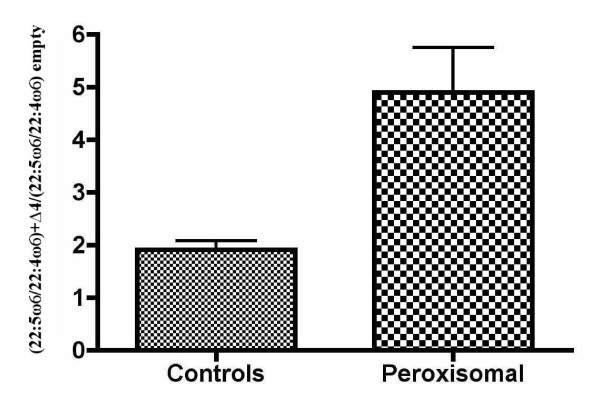
**Omega-6 effects of transfection with Δ4-desaturase on grouped controls and patients with the Zellweger syndrome**. This figure displays the number of times that the 22:5ω6/22:4ω6 ratio increased when adding the Δ4-desaturase, in normal children, as well as in the ZS patients. A new index was obtained by dividing the 22:5ω6/22:4ω6 ratio with the Δ4-desaturase added by the same ratio without the enzyme [(22:5ω6/22:4ω6)+Δ4/(22:5ω6/22:4ω6) empty]. Bars are mean values ± SEM. Despite the effect of grouping patients and controls by unpaired t-tests, the statistical significance was still very high (p = 0.0009).

Some of these changes did not reach statistical significance when all the cases were grouped in controls and ZS patients (tables 2 and 3) because any individual variation was masked by grouping. This is especially true for the 22:5ω6/22:4ω6 ratio, whose variability was the largest, as can be seen in fig. 4. On the whole, though, these data show that, starting at lower values in the ZS patients, Δ4-desaturation of both ω3 and ω6 long PUFAs normalized after expressing the gene (tables 2 and 3 and Fig 3 and 4). This is better illustrated in Fig. 5 and 6, which compare the number of times that the 22:6ω3/22:5ω3 and 22:5ω6/22:4ω6 ratios, respectively, increased in controls and ZS patients (see legends to the figures). By expressing the results that way, even grouping did not mask the very significant differences between controls and ZS patients, which were larger in the case of the 22:5ω6/22:4ω6 ratio.

## Discussion

Currently, the Δ4-desaturation pathway has been disregarded as an operative route in mammals. This has mainly been due to the fact that the corresponding enzyme has never been characterized. Also, the finding that DHA is decreased in Zellweger-syndrome patients [13] promoted the study of an alternate route linking the defective VLCFA β-oxidation in ZS patients to their DHA deficiency [28]. This is a very attractive concept, and is consistent with the fact that 24:6ω3 is converted to DHA when β-oxidation of VLCFA is intact, which is not the case in peroxisomal-disorder patients. That DHA is readily produced from 24:6ω3 is an incontestable fact, as we have checked by adding this very long PUFA (VLPUFA) to cells in culture (data not shown). However, this requires adding substantial amounts of 24:6ω3, while this PUFA is normally present only in trace amounts in human cells. Whether or not this is due to rapid metabolism of 24:6ω3 (and its counterpart of the ω6 family 24:5ω6) remains to be demonstrated. A recent paper [29] suggests that both are simple runaway elongation by-products. Yet, even if these VLPUFAs were the main (or even the only) route for the synthesis of DHA (and 22:5ω6), the present paper shows that, by transfecting the gene that codes for Δ4-desaturase (*Fad4*), this classic, putative Δ4-desaturation route is also operative in the human species.

This may be important for patients within the Zellweger syndrome spectrum (ZS, NALD, IRD). Indeed, if DHA deficiency is a primary defect in these patients, normalization of its biosynthesis could cause even more beneficial effects than treatment with DHA alone. Even if quantitatively small, we do not know if endogenous DHA synthesis in the liver is of prevalent importance in the healthy human. Some data in the rat suggest so [30]. Normalizing plasma and erythrocyte DHA levels in ZS patients is quite easy, usually also leading to an increase in plasmalogen synthesis and in VLCFA β-oxidation, together with marked improvements in liver function tests [23]. However, once normalized, maintaining the right levels is quite critical, especially with the very high doses of ω3 PUFAs that are currently recommended. This may easily lead to PUFA imbalance, with an undesirable and dangerous decrease in the ω6 PUFAs, mainly arachidonic acid (AA, 20:4ω6). AA is an important precursor in prostaglandin and leukotriene biosynthesis and there may be other abnormalities, whose consequences we do not currently know. It must also be pointed out that most of the triglyceride DHA preparations commercially in use nowadays have linoleic acid (18:2ω6) added in the form of sunflower oil. This neutralizes the effects of DHA and may also add to the blockage of AA biosynthesis by an excess of precursor. Besides, giving too much DHA may block its own biosynthesis, by negative feedback caused by an excess of the product. These facts are not normally taken into account, and DHA is lately widely recommended without even looking at the subject's fatty acid changes.

In summary, this paper shows that adding Δ4-desaturase to human cells greatly increases the production of both DHA and 22:5ω6 (DPA). The latter is known to increase in DHA deficiency as a compensatory mechanism, since its biochemical structure is very similar to DHA. This VLPUFA was also diminished in the ZS patients, and its increase when we added the Δ4-desaturase construct supports the view that this route is functional for both series of PUFAs. Therefore, the changes obtained seem to be more physiological than simply adding DHA to the diet. Even if the conditions we used were "in vitro" experiments in human EBV immortalized lymphocytes, the results were of an extremely high statistical significance, and we believe that they warrant the continuation of this work. Perhaps introducing Δ4-desaturase into these patients by genetic engineering could lead to normalization of the DHA (and 22:5ω6) biosynthesis in patients with the Zellweger syndrome and other peroxisome biogenesis disorders. This may be highly beneficial for these patients.

## Conclusions

This paper shows for the first time that, by transfecting human cells with the gene coding for Δ4-desaturase (*Fad4*), these cells can express the enzyme as lower eukaryotes do. This leads to an increased biosynthesis of DHA and DPA in healthy subjects, and to normalization of the DHA deficiency in Zellweger's syndrome, together with an increase in DPA. Although this is only an "in vitro" study in human cells, it opens new avenues for research on human fatty acid metabolism and the possibility of devising a new treatment for these life-threatening diseases.

## Methods

### Human subjects

Blood samples from 14 children with generalized peroxisomal disorders and 16 normal controls were used for the present study. The age range was similar in both cases (3 months to 12 years, for peroxisomal disorders, and 2 months to 14 years, for controls). The clinical severity of the peroxisomal-disorder patients was highly variable, including two cases with classic Zellweger's syndrome and 12 cases within the neonatal adrenoleukodystrophy/infantile Refsum's disease (NALD/IRD) spectrum. Control samples were obtained from normal children in the occasion of blood drawings performed for routine analyses. They had no abnormalities, with the exception of a small baby with a suspect diagnosis of non-ketotic hyperglycinemia, who could not be analytically distinguished from the rest of controls. Parental consent was obtained in all cases. Peripheral blood was drawn after a minimum of 6 hours of fasting and collected in EDTA tubes. The preferred volume was 3 ml, but in small children, as little as 1 ml was used. Processing of samples was started immediately in all cases. Never were the cells or plasma frozen before starting the procedure, since it was found that some PUFAs may be degraded by freezing and thawing, especially in erythrocytes. Centrifugation was carried out at 2330 × g for 10 minutes. Plasma was removed, and the buffy coat and some adjacent red blood cells and remaining plasma were collected. The process was repeated after re-suspending and washing the erythrocyte pellet with about 5 ml of saline solution, trying to completely recover the white cell layer. A Lymphocyte Isolation Solution Gradient (Rafer) was used to isolate peripheral blood mononuclear cells (PBMC) from the white cell layer, according to the manufacturer's instructions. The fatty acid composition of the removed plasma and washed erythrocytes was separately determined as the routine control of the patients (data not shown), as well as to check the fatty acid status of the normal subjects.

### Establishment of the B lymphoblastoid cell lines (BLCL)

Lymphoblastoid cell lines were established by Epstein-Barr Virus (EBV) transformation/infection of peripheral blood mononuclear cells (PBMC) in the presence of phytohemagglutinin (PHA) [31]. PBMCs were incubated with B95-8 cell supernatant (containing Epstein-Barr virus (EBV) in the presence of 10 μg/mL of PHA in RPMI1640 supplemented with 20% (v/v) heat inactivated, fetal calf serum (FCS), 2 mM of L-Glutamine, 100 units/mL of penicillin, 100 μg/mL of streptomycin and 0.5 μg/mL of Amphotericin B (Gibco, Invitrogen). Cells were plated on 24-well plates and were incubated at 37°C, 5% CO2 until the growth of the B lymphoblastoid cell lines (BLCL) was established. Thereafter, cells were expanded and routinely grown in RPMI1640 culture medium supplemented with 10% heat inactivated FCS, 2 mM of L-Glutamine, 100 units/mL of penicillin and 100 μg/mL of streptomycin.

### Generation of the pM2Δ4 vector

The open reading frame of the *Thraustochytrium sp *Δ4 desaturase (*Fad4*) was identified and cloned from a cDNA library of *Thraustochytrium sp*[27]. To generate pM2Δ4, the cDNA fragment was released by a BamHI/EcoRI double digestion and inserted into the BglII/EcoRI double digested eukaryotic expression vector pM2 under the control of the constitutive SV40 early promoter.

### Generation of pIRES2EGFP-FlagΔ4 vector

Preliminary experiments were performed co-trasfecting the pM2Δ4 or empty pM2 together with the pIRES2 EGFP vector as a control for efficency of transfection. Initially, Lipofectamine LTX (Invitrogen) was used as the transfection reagent. This produced a slight increase in the desaturase activity in the pM2Δ4 transfected cells with respect to the ones transfected with the empty pM2 vector. However, with these conditions, the Δ4-desaturase activity was too small to reach statistical significance. Moreover, the fatty acid composition of the cells was affected by the use of lipofectamine LTX, as shown in the empty pM2 vector transfected cells (data not shown). To circunvent this, electroporation was chosen as the method for transfection.

To better monitor the transfection efficiency, the coding sequence of *Fad4 *was cloned into the pIRES2EGFP vector. The mammalian expression vector pIRES2EGFP contains an Internal Ribosome Entry Site (IRES) between the MCS and the EGFP coding region that would permit both the *Fad4 *and the EGFP to be translated from a single bicistronic mRNA. Moreover, it also contains the Human cytomegalovirus (CMV) immediate early promoter. The CMV promoter has been shown to be stronger in B lymphoid cell lines than the SV40 promoter [32]. To further increase the translation efficiency in eukaryotic cells, a Kozak consensus translation initiation site was included. A flag sequence was added to allow the detection of the expressed FAD4 desaturase in the transfected cells. To achieve that, the *Thraustochytrium sp *Δ4 cDNA was amplified by PCR using the plasmid pM2Δ4 as a template. Restriction sites were added to the 5' ends of both PCR primers to allow further cloning. The forward primer was designed to contain a BglII restriction site (underlined), a Kozak consensus sequence, the ATG start codon (bold), and a Flag tag sequence (italic), as follows:

5'-AAAGATCTCCACC**ATG***GACTACAAGGACGACGATGACAAG*GGCAGCGGCACGGTCGGCTACGACGAG-3'. Reverse primer contained a EcoRI site (underlined) and a stop codon (bold), and it was:

5'-AGGAAT**TC****A**GGCAGCGCGCTGC-3'. The PCR reaction was performed using Phusion High-Fidelity DNA Polymerase (Finnzymes). The conditions were: an initial denaturation of 1 min. at 98 °C, followed by 30 cycles of 10 s at 98°C, 12 s at 54°C, and 1 min at 72°C, and a final extension of 7 min at 72°C. The 1.6 kb PCR-amplified DNA fragment was isolated by electrophoresis using 0.8% agarose, gel-purified using NucleoSpin Extract II (MACHEREY-NAGEL), and, following digestion with BglII and EcoRI restriction enzymes (Takara), cloned into BglI/EcoRI-digested pIRES2EGFP to generate the construct pIRES2EGFP-FlagΔ4. To rule out the presence of PCR-generated mutations, the entire insert and flanking regions were sequenced using the BigDye^® ^Terminator v3.1 Cycle Sequencing Kit (Applied Biosystems), and an Applied Biosystems Model 3730 DNA Analyzer. The CodonCode Aligner software was used for alignment and analysis of the sequences. The coding sequence was identical to that in pM2Δ4.

### Expression of FlagΔ4-desaturase protein

To confirm the functionality of the pIRES2EGFP-FlagΔ4 vector, the recombinant plasmid was used to transfect the colon cancer cell line HCT116, and overexpression of the 60 kDa Flag-FAD4 was confirmed by Western blotting (data not shown). Functional activity of the Δ4-desaturase was not affected by the Flag tag, since the pIRES2EGFP-FlagΔ4 construct restored desaturase activity to the same extent as a similar construct without the tag (pIRES2EGFP-Δ4) (data not shown).

### Transfection of the B lymphoblastoid cell lines

Plasmid pIRES2EGFP-FlagΔ4 was used to transfect BLCLs. 40 μg of DNA were electroporated at 250 V and 950 μF into 6-10 × 10^7 ^cells in 0.4 cm cuvettes (Gene Pulser × cell, BioRad). Cells were plated into 6-well plates and grown in RPMI-1640 medium supplemented with 20% FCS. In each case, electroporation of pIRES2EGFP was done in parallel and used as a control. On day 3 after transfection, EGFP expression was confirmed using an inverse fluorescence microscope (Leica DM IL LED), and alive cells were purified by centrifugation upon Lymphocytes Isolation Solution Gradient (Rafer). A small fraction was separated for protein quantification, and the rest of the cells were pelleted and subjected to fatty acid analysis. Protein quantification was carried out using the BCA Protein Assay Kit (Pierce) with Bovine serum albumin (BSA) as the protein standard.

### Fatty acid analysis

The final lymphocyte pellet was directly used for fatty acid analysis. In our experience, measuring the total fatty acid content of a sample is the best to evaluate its PUFA composition. This allows direct methanolysis of the sample, and loses are minimized.

Thus, total lipids (and plasmalogens) were directly transmethylated from the washed lymphocyte pellet with HCl-methanol, basically by the method of Lepage and Roy [33] with slight variations [14]. In order to check the linearity of the flame ionization detector (FID) in each analysis, two internal standards of widely different molecular weights (13:0 and 23:0) were used instead of one. Only when the areas of the two internal standards were close enough (within ≤ 10% variation) were the results considered acceptable.

Briefly, 1.95 ml of methanol-benzene 4:1 (v/v) and 50 μl of the internal standard mixture (in the same solvent), containing 25 μg of each internal standard, was added to the cell pellet in a methanolysis, Corning tube. After slowly adding 0.2 ml of acetyl chloride while continuously vortexing the tube, the tightly stopped tube was placed in a dry bath at 100°C for an hour. After cooling, 5 ml of 6% K_2_CO_3 _was added to neutralize the acid. After centrifuging at 2330 × g for 10 minutes, the clear supernatant was ready for direct injection into the gas chromatograph. When the protein content of the sample was too low, the supernatant was concentrated under a stream of N_2 _in a small glass tube and injected immediately. Never was the concentrate stored in the freezer or refrigerator for later use, since this was found to decrease the PUFA levels.

The total fatty acid methyl esters (FAME) were separated on a 30 m long, 0.25 mm ID RTX-2330 capillary column, programmed between 140°C and 200-220°C, at variable rates (2°-4° C/minute) according to the state of the column, until the whole spectrum of fatty acids was resolved. When necessary, a second RTX-2330 and a 30 m long 0.2 mm ID, BPX70 column was used to check the results. Three different Hewlett Packard gas chromatographs were used for these columns: one 6890 and two 5890-II. The carrier gas was helium, at rates of 0.8-1.2, depending on the column type and state. Injector and detector temperatures were 250°C and 260°C, respectively. When necessary, the identity of some peaks of interest was confirmed by mass spectrometry using a Hewlett Packard, 5970B mass spectrometer. Quantitation of fatty acids was performed with Merck Hitachi, 2000 and 2500, computer-integrators. Quantitative data were referred to mg of protein. However, percent values were preferred, due to the large protein variability of the cells.

### Statistical analysis

The statistical differences were analyzed by Student t-tests. Two types of analyses were performed, paired and unpaired t tests. To estimate the Δ4-desaturase activity we used simple product/precursor ratios, that is, 22:6ω3/22:5ω3 for DHA synthesis, and 22:5ω6/22:4ω6 for formation of the ω6 docosapentaenoic acid (DPA). These two ratios were evaluated in the cells transfected with the empty plasmid (pIRES2EGFP) and in those transfected with the Δ4 construct (pIRES2EGFP-FlagΔ4). To diminish the individual variability a paired t-test was used for this evaluation (Fig. 3 and 4). Unpaired t-tests were used for grouping comparisons between patients and controls (Tables 2 ad 3, and Fig. 5 and 6). GraphPad Prism 4 and t-test calculator (GraphPad Sofware) were used as the software.

## Competing interests

The authors declare that they have no competing interests.

## Authors' contributions

MM devised the original project on fatty acid synthesis and its possible impact on Zellweger-syndrome patients, performed all fatty acid analyses, evaluation and statistics, and wrote most of the manuscript. NI carried out the molecular biology and transfection studies, and participated in the establishment of the B lymphoblastoid cell lines, in the design of the molecular part of the study, and in the drafting of the manuscript. FS participated both theoretically and manually in the establishment of the B lymphoblastoid cell lines. ND performed the preliminary transfection experiments and provided ideas regarding the molecular methods to be used. XQ discussed with MM the original project and prepared the Fad4 plasmid from *Thraustochytrium sp*. WR provided the original pM2Δ4 construct for its use in mammals and participated in helpful discussions about the project. All authors read and approved the final manuscript.
